# Molecular Characteristics, Prognostic Value, and Immune Characteristics of m^6^A Regulators Identified in Head and Neck Squamous Cell Carcinoma

**DOI:** 10.3389/fonc.2021.629718

**Published:** 2021-03-18

**Authors:** Xiuchao Geng, Yuhao Zhang, Zhaomu Zeng, Zhongrui Zhu, Hong Wang, Wentao Yu, Qiang Li

**Affiliations:** ^1^ Faculty of Integrated Traditional Chinese and Western Medicine, Hebei University of Chinese Medicine, Shijiazhuang, China; ^2^ Hebei Key Laboratory of Chinese Medicine Research on Cardio-cerebrovascular Disease, Hebei University of Chinese Medicine, Shijiazhuang, China; ^3^ Department of Neurosurgery, Affiliated Hospital of Hebei University, Baoding, China; ^4^ School of Clinical Medicine, Hebei University, Baoding, China; ^5^ Faculty of Acupuncture-Moxibustion and Tuina, Hebei University of Chinese Medicine, Shijiazhuang, China

**Keywords:** m^6^A regulators, IGF2BP2, head and neck squamous cell carcinoma (HNSCC), pan-cancer, prognosis, immune cell infiltration

## Abstract

N6-methyladenosine (m^6^A) plays crucial roles in a diverse range of physiological and pathological processes, and it is believed that it tremendously promotes neoplasia and progression. However, knowledge of the molecular characteristics of m^6^A modification, its prognostic value, and the infiltration of immune cell populations in head and neck squamous cell carcinoma (HNSCC) is still insufficient. Therefore, a pan-cancer genomic analysis was systematically performed here by examining m^6^A regulators at the molecular level within 33 multiple cancer types, and the correlations between the expression of m^6^A molecules were researched using datasets from The Cancer Genome Atlas (TCGA). Based on the above analysis, insulin-like growth factor 2 mRNA-binding protein 2 (IGF2BP2) is upregulated in HNSCC and may serve as an independent prognostic factor of overall survival, thus showing potential as a prognostic biomarker in HNSCC. Genetic alteration analyses elucidated the reasons for the abnormal upregulation of IGF2BP2 in HNSCC. As a result, IGF2BP2 was selected for further univariate and multivariate analyses. The functions of the related genes were annotated through gene set enrichment analysis, and the activation states of multiple biological pathways were shown by gene set variation analysis. We found that LRRC59 and STIP1 may act as IGF2BP2-associated genes to have a regulatory function in the m^6^A modification. In addition, we found that the status of immune cell infiltration was correlated with the level of IGF2BP2 gene expression. Our results provide supplementation at the molecular level for epigenetic regulation in HNSCC and insight into effective immunotherapy targets and strategies.

## Introduction

The classic epigenetic modifications are DNA methylation, histone modification, and chromatin remodeling, which take part in many basic biological activities of carcinogenesis, including malignant development, and are associated with the prognosis of various kinds of cancer, including head and neck squamous cell carcinoma (HNSC, HNSCC) (1). In the past, reports on tumor methylation modifications have concentrated mostly on DNA methylation and offered potential biomarkers for cancer diagnosis (2). In addition to these traditional epigenetic modifications, mRNA modification is known as another epigenetic regulation of gene expression. Its dynamic regulation and disorder are closely related to translation control, RNA splicing defects, and the occurrence, maintenance, and progression of various tumors (3, 4). The degree of RNA modification is highly sensitive to changes in the cell microenvironment or the transformation of physiological states, and RNA modification changes will in turn influence cell regulation and adaptation (5). Among the types of RNA modifications, N6-methyladenosine (m^6^A) is recognized as the first and most common modification in eukaryotic mRNA (6).

m^6^A modification has been studied for 40 years. As a posttranscriptional modification with dynamic and reversible characteristics, m^6^A methylation has a profound impact on gene regulation (7), and its biological importance has also been emphasized by recent research progress. m^6^A modification participates in numerous basic biological processes, such as mRNA stability, mRNA translation, RNA splicing, and the phase separation potential of mRNA (8–10). Many physiological processes of the human body are closely related to m^6^A, such as spermatogenesis (11) and spermatogonial differentiation (12), hematopoietic stem cell development (13), and antiviral innate immunity (14). Moreover, a disturbance in m^6^A modification is associated with the pathogenesis of diverse cellular processes, including reduced cell proliferation, impaired self-renewal ability, developmental defects, and cell death (15). m^6^A modification has crucial functions in many human diseases, including infertility (16), nervous system diseases (17), early embryonic retardation (18), immune diseases, and multiple cancers (19). Currently, m^6^A modification, especially the role of its regulatory enzymes (writers, erasers, and readers), in cancer biology has been recognized as a prominent hot spot in terms of tumorigenesis, malignant progression, and potential biological target screening. Writers form a multisubunit methyltransferase complex to upregulate the level of m^6^A, while erasers are m^6^A demethylases, making this event reversible. Readers can decode m^6^A methylation information and convert it into effectors of functional signals. Recently, increasing evidence has shown that changes in m^6^A regulators can promote the development of several cancers (20), including glioblastoma, breast and cervical cancers, liver and gallbladder cancers, and bladder and prostate cancers (21–23). However, despite some progress in revealing their role in multiple cancers, the characteristics of m^6^A modification in HNSCC are still very inadequate.

HNSCC is a highly invasive malignant tumor that originates from the mucous membrane of the hypopharynx, larynx, oral cavity, and oropharynx, accounting for 90% of all head and neck cancers (24). HNSCC has more than 650000 confirmed cases worldwide, causing more than 300000 deaths every year (25) and ranking as the sixth most common and fatal cancer throughout the world (26). Although much progress has been made in the treatment methods (surgery, chemotherapy, and radiotherapy), the clinical prognosis of HNSCC patients is still poor. The 5-year survival rate of patients is still approximately 50%, which remains to be improved, and suggests that approximately 30-50% of HNSCC patients will experience local recurrence and distant metastasis (24). Therefore, the search for specific molecular markers of HNSCC is of great significance for better understanding of its progress and for developing new therapeutic methods. At present, an increasing number of studies have concentrated on the mechanism of pathogenesis, especially genetic and epigenetic events, at the molecular level (27). Currently, m^6^A modification has been reported to play a vital function in cell infiltration in the tumor microenvironment, and the m^6^A modification pattern can be used to help identify different immune phenotypes in gastric cancer (28). In addition, HNSCC was one of the first diseases for which immunotherapy was developed, which has greatly changed the therapeutic prospects of cancer patients. However, until now, there have been few reports about the relationship between m^6^A methyltransferase and clinicopathological features, its prognostic value, and the infiltration of immune cells in HNSCC. Accordingly, the function of m^6^A modification in tumor diagnosis, prognosis, and immune cell infiltration in HNSCC remains to be explored and could provide treatment targets for HNSCC.

As a result, we investigated the expression modes of 24 m^6^A regulatory genes in 33 cancers and explored the differential expression of IGF2BP2 in HNSCC and its clinical significance. Based on the clinical and sequencing data of The Cancer Genome Atlas (TCGA)-HNSCC cohort, the relationships among differential expression, genetic alterations, and clinicopathological features, including survival, were analyzed. Gene set enrichment analysis (GSEA) and gene set variation analysis (GSVA) were used to analyze the molecular mechanism of IGF2BP2 abnormalities in HNSCC. On the other hand, the characteristics of m^6^A genes, including IGF2BP2, were analyzed to examine their relationship with immune cell infiltration in HNSCC. The purpose of this study was to provide supplementation at the molecular level for epigenetic regulation in HNSCC and ideas for effective immunotherapy targets and strategies.

## Materials and Methods

### Acquisition of Datasets and Data Preprocessing

For datasets in the TCGA, the R package TCGAbiolinks (29) was used to download RNA sequencing data and full clinical annotation information from the Genomic Data Commons (GDC, https://portal.gdc.cancer.gov/) and was specialized for an integrated analysis of GDC data (29). Patients for whom survival information was lacking were excluded from further evaluation. Information on somatic mutation data for HNSCC patients was obtained from the TCGA database as well. The expression of m^6^A regulatory genes in 33 cancers was analyzed with the TIMER2.0 database (http://timer.comp-genomics.org/) (30), in which the Wilcoxon test (also named the Mann–Whitney test) was applied to evaluate the P value. Sequencing and copy number variation (CNV) data provided by cBioPortal (http://www.cbioportal.org/) for cancer genomics were used to detect the genetic alterations in m^6^A regulatory proteins within 504 HNSCC patients/samples. IGF2BP2 mRNA expression, somatic mutations, CNVs, and the level of methylation in HNSCC were analyzed with UCSC Xena (https://xenabrowser.net/heatmap/). Twenty-four interactions between m^6^A regulators were elucidated with the help of the STRING database (http://www.string-db.org/). Immune cell infiltration was analyzed using the TIMER2.0 database.

### Prognostic Value and Survival Analyses

Univariate and multivariate Cox regression analyses were used to assess the prognostic value of m^6^A regulators through their expression pattern in the TCGA. Both the “survival” and “survminer” (https://CRAN.R-project.org/package=survminer) R packages were used to investigate every gene. Four hundred and twenty-nine HNSCC patients were divided into different subgroups (high and low groups) by their characteristics and median gene expression values.

### Differential Expression Analyses

The TCGAbiolinks R package was used to download TCGA-HNSC read count data. Patients with HNSCC were assigned to high and low IGF2BP2 groups according to the median cutoff value. A total of 11,297 long noncoding RNAs (lncRNAs) were ultimately screened for differential expression analyses. The DEseq2 R package (version 1.26.0) in R software (https://www.r-project.org/) was used to perform data standardization and calculate differences in the expression data. An absolute log2-fold change (FC) > 1 and p-value < 0.05 were considered the criteria for the differential expression of lncRNAs. The ggplot2 R package was used to draw a volcano plot.

### Gene Set Cancer Analysis (GSCA) Lite Analysis

The differential expression profiles and survival outcomes of 24 m^6^A regulator genes among 33 cancer types were assessed using the GSCALite web server (31). Only 14 cancer types (THCA, KIRP, BLCA, LIHC, HNSC, BRCA, LUAD, PRAD, ESCA, KICH, LUSC, KIRC, STAD, and COAD) had paired samples (paired tumor-normal tissue) with available expression data. GSCALite was also used to study the effect (activation or inhibition) of m^6^A molecules on pathways related to cancer. In GSCALite, the scores of 7876 samples, 10 cancer-related pathways, and 32 cancer types (acute myeloid leukemia (AML) was excluded) were obtained from reversed-phase protein array (RPPA) data from The Cancer Proteome Atlas (TCPA). A total of ten cancer-related pathways, including apoptosis, PI3K/AKT, and TSC/mTOR, were identified, and m^6^A regulators that impacts (activate or inhibit) at least five different cancers were revealed by GSCALite.

### Gene Set Enrichment Analysis (GSEA)

GSEA was performed with the JAVA program of the MSigDB v6.1 acquired from the website of the Broad Institute (http://software.broadinstitute.org/gsea/) (32). Then, we applied GSEA with standard settings as implemented in the R package clusterProfiler to assign pathway activity estimates to each sample. Finally, 18419 genes were included in the GSEA program, where “c2.cp.kegg.v7.1.symbols.gmt” was regarded as the hallmark gene set (33). A normalized p-value < 0.05 and a false discovery rate < 0.25 of the hallmark gene set were considered significantly enriched.

### Gene Set Variation Analysis (GSVA) and Functional Annotation

GSVA was performed with the “GSVA” R package to determine the differences in physiological processes based on m^6^A modification patterns. GSVA is widely employed as a method to estimate variation in signaling pathways and physiological processes within a nonparametric and unsupervised method for samples of an expression dataset (34). Thus, the gene set “c2.cp.kegg.v7.1.symbols.gmt” was accessed through the MSigDB for GSVA. An adjusted p-value < 0.05 was considered statistically significant.

### The Human Protein Atlas

The Human Protein Atlas (https://www.proteinatlas.org/) provides pathological specimens for the immunohistochemical analysis of prognostic RNAs, such as m^6^A methyltransferases. A survival analysis of associated genes in HNSCC was also performed. Information on the patients, staining intensity, and quantity can be obtained online.

### Statistical Analysis

SPSS 20.0 (SPSS, Chicago, IL), R software V3.6.2 and GraphPad Prism 8 (GraphPad Software, Inc., La Jolla, CA, USA) were applied for statistical analysis. Continuous variables are presented as the mean ± standard deviation. Univariate and multivariate Cox regression analyses were used to illustrate their prognostic value and associations with clinical characteristics at the molecular/pathological level. Kaplan–Meier curves with a two-sided log-rank test were used to compare the overall survival (OS) of the patients in the high and low expression groups. p-values < 0.05 were considered statistically significant.

## Results

### Gene Expression Landscape of m^6^A Regulators

**Figure 1:**
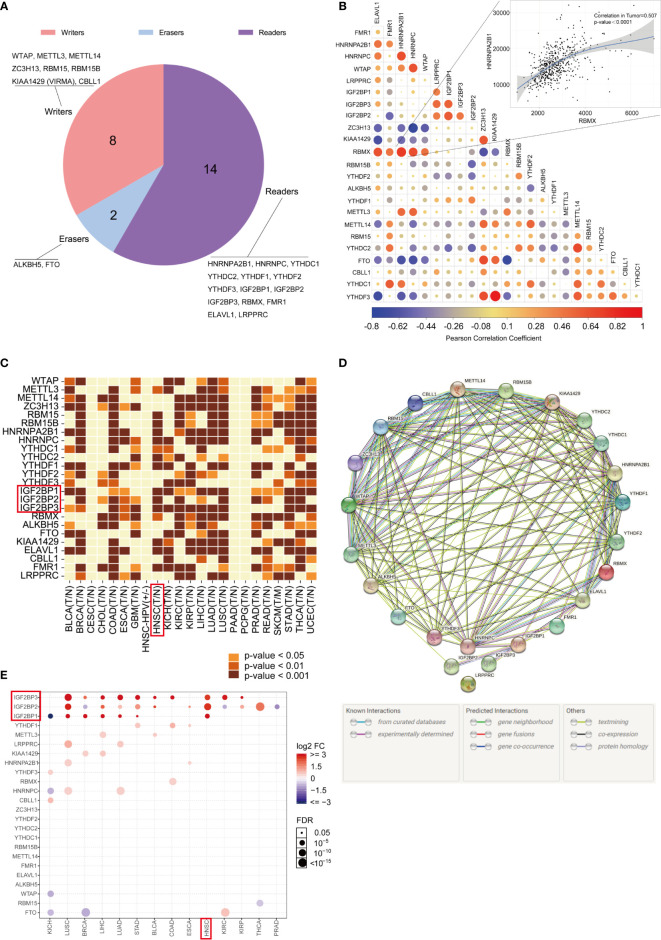
Gene expression landscape of m^6^A regulators in pan-cancer. **(A)** The proportions of readers, writers, and erasers among m^6^A regulators. **(B)** Correlations between the expression of 24 m^6^A regulators. The correlation between RBMX and HNRNPA2B1 is illustrated as a scatter plot (Pearson correlation). A positive correlation is indicated in red, and a negative correlation is indicated in blue. The color intensity and size of the circle are proportional to the correlation coefficient. **(C)** The expression profiles of the 24 m^6^A regulatory genes in the TCGA pan-cancer dataset originating from the TIMER database. **(D)** The interactions among 24 m^6^A regulatory genes (STRING). **(E)** GSCALite was used to analyze the gene expression profiles of 24 m^6^A regulatory genes in 14 types of cancer.

The genome-wide omics data, consisting of more than 10,000 human samples of 33 cancer types, were acquired from the TCGA for analyses (**Supplementary Material: Table S1**). The literature was reviewed, and a catalog of 24 genes that function mainly as regulators of m^6^A modifications was curated here: 8 writers (WTAP, METTL3, METTL14, ZC3H13, RBM15, RBM15B, KIAA1429 (VIRMA), and CBLL1), 2 erasers (ALKBH5, FTO) and 14 readers (HNRNPA2B1, HNRNPC, YTHDC1, YTHDC2, YTHDF1, YTHDF2, YTHDF3, IGF2BP1, IGF2BP2, IGF2BP3, RBMX, FMR1, ELAVL1, and LRPPRC). The proportions of readers, writers, and erasers among m^6^A regulators are listed in **Figure 1A**. The correlations between the expression of 24 m^6^A regulators in the TCGA-HNSC cohort were determined by Pearson correlation analysis, and the results are displayed in **Figure 1B**. The TIMER database was used for analysis, and we found that m^6^A regulators were abnormally expressed in cancers in a cancer-specific pattern. Compared with their paired normal controls, RBM15, METTL3, and IGF2BP1/2/3 were obviously upregulated in the TCGA-HNSC dataset, while WTAP, METTL14, ZC3H13, YTHDF2, YTHDF3, ALKBH5, FTO, CBLL1, and LRPPRC were not upregulated (**Figure 1C**). To better visualize the interactions between the 24 m^6^A regulators, their relationships were determined with the STRING database (**Figure 1D**). The GSCALite web server was used, and we found twenty-four m^6^A regulators to be differentially expressed in 14 cancer types (**Figure 1E**). We found that IGF2BP1/2/3 were upregulated in HNSCC, consistent with the data from TIMER.

### Relationships Between m^6^A Regulatory Genes and Pan-Cancer

Among the different types of cancer, the prevalent genetic and expression alterations in m^6^A regulators could largely give rise to the breakthrough of translational medicine, as m^6^A regulatory genes were closely related to patient OS across 33 cancer types in our study. It was also implied by our results that 24 m^6^A regulatory genes were related to the OS of patients with at least one cancer type, where some of the genes, including IGF2BP1/2/3, showed oncogenic features, and their increased expression was related to worse survival in patients with various cancers. In particular, upregulation of the IGF2BP2 gene was associated with poor patient survival in seven cancer types, including HNSCC (**Supplementary Material: Figure S1A**).

To further explore the underlying mechanisms by which m^6^A regulatory genes participate in multiple cancers at the molecular level, the effects (activation or inhibition) of m^6^A regulatory genes on pathways accompanying cancers were analyzed with GSCALite. It was found that m^6^A regulatory genes are functionally expressed and are related to oncogenic activation or inhibitory pathways (**Supplementary Material: Figure S1B**). A large number of activated pathways, namely, the cell cycle, epithelial-mesenchymal transition (EMT), DNA damage response, PI3K-AKT, RTK, and RAS/MAPK, were found to be associated with the expression of IGF2BP2, RBMX, and HNRNPC. These results confirmed that m^6^A regulators are functionally related to multiple cancers.

### Single Nucleotide Polymorphism (SNP) Mutations in m^6^A Regulatory Genes

We found that single nucleotide polymorphism (SNP) occupied the first place in variant type (**Supplementary Material: Figure S2A**). SNP analysis showed that missense mutations were the most frequent variants in the m^6^A-regulatory genes (**Supplementary Material: Figure S2B**). The most frequent SNV type was C > T (**Supplementary Material: Figure S2C**).

In addition, we summarized the incidence of single nucleotide variants (SNVs) in 24 m^6^A regulators in 33 pan-cancer samples with GSCALite. Among 1443 samples, 1002 harbored mutations in m^6^A regulators, with a frequency of 69.44%, among which ZC3H13 showed the highest mutation frequency, followed by KIAA1429 (**Supplementary Material: Figure S2D**).

### The Prognostic Role of m^6^A Regulators and Their Correlations With Clinical Characteristics in HNSCC

**Figure 2:**
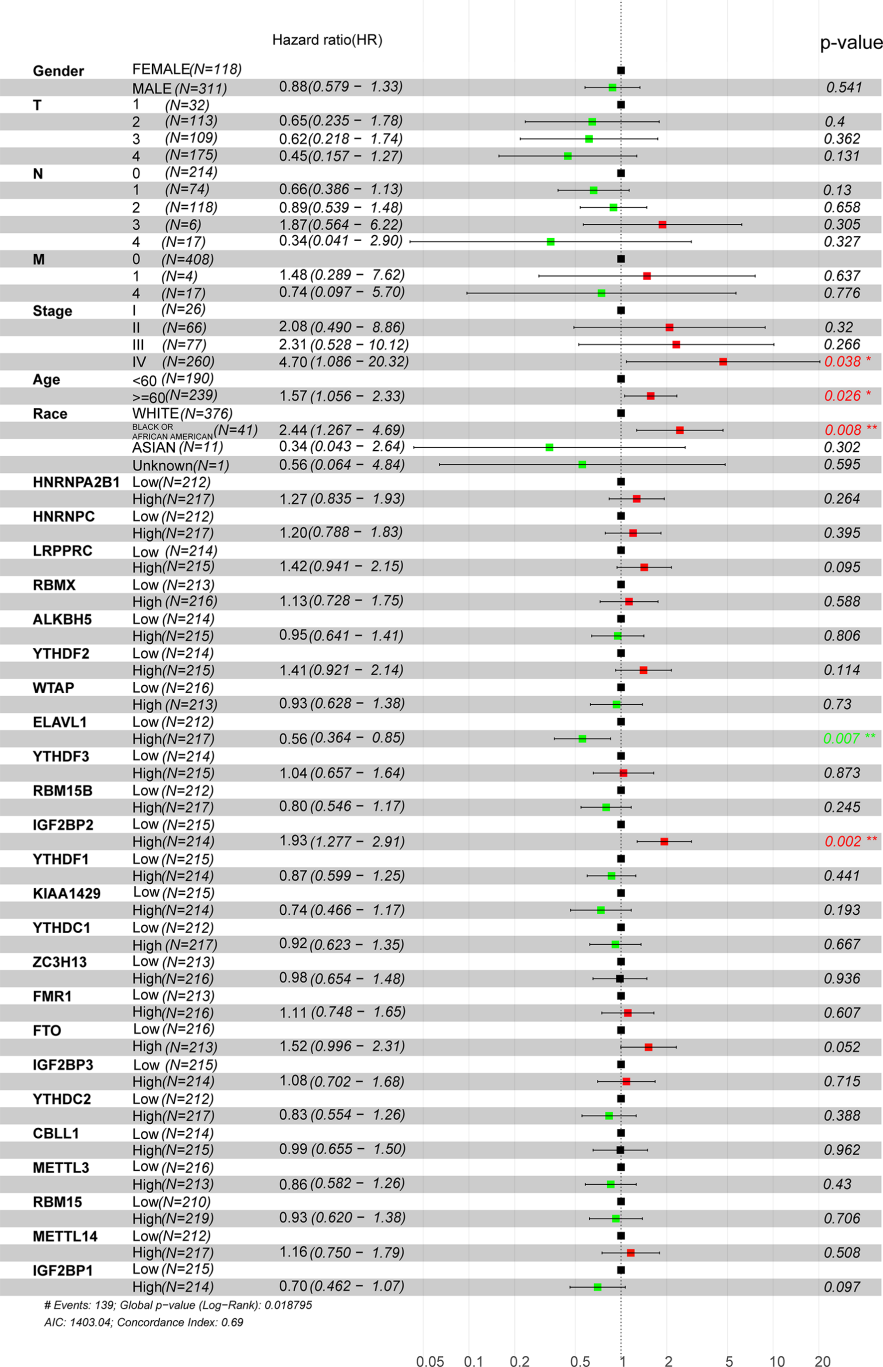
Univariate Cox regression analysis of m^6^A regulators in HNSCC patients in the TCGA dataset. Tumor node metastasis classification: T (tumor), N (lymph node), and M (metastasis).

Considering the relationship between m^6^A methyltransferases and the malignant progression of HNSCC, univariate and multivariate survival analyses were performed to further determine the prognostic value of m^6^A methyltransferases in HNSCC using Cox proportional hazards models of their expression levels in the TCGA dataset. A total of 429 HNSCC patients were assigned to two groups (high and low) based on the median expression value of each gene. Stage IV disease and high LRPPRC and IGF2BP2 expression levels were shown to be independent prognostic factors for OS. Multivariate analysis showed that patients aged ≥60 years, with stage IV disease, of African American race, and high ELVAL1 and IGF2BP2 expression were risk factors for poor OS (hazard ratio (HR)> 1) (**Figure 2**). As shown in **Supplementary Material: Table S2**, IGF2BP2 was correlated with T-stage based on the median cutoff of IGF2BP2 (P = 0.023). However, IGF2BP2 did not correlate with clinical characteristics such as age, race, stage, or sex. ELVAL1 was correlated with the clinical characteristic race (P = 0.014). LRPPRC was correlated with the clinical characteristics sex (P = 0.016), T-stage (P = 0.044), and race (P = 0.040) (**Supplementary Material: Table S3**).

### The Expression and Prognostic Value of IGF2BP2

**Figure 3:**
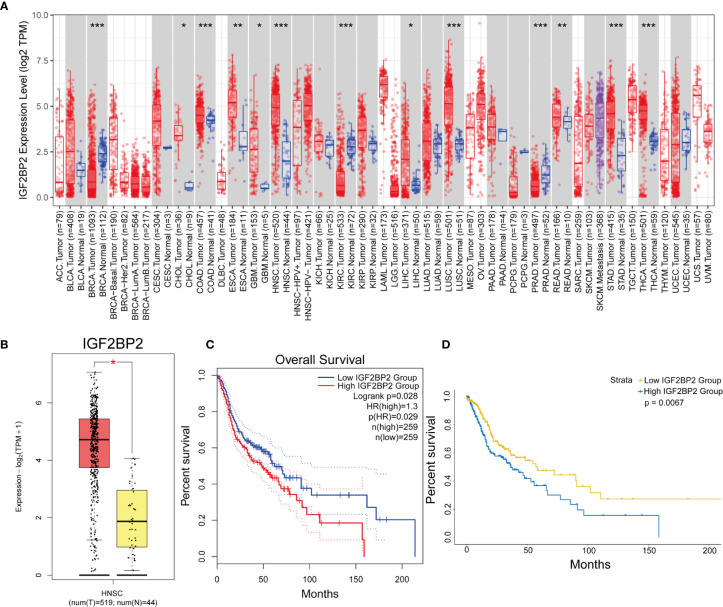
The expression and prognostic value of IGF2BP2 in HNSCC. **(A)** The expression of the IGF2BP2 gene in pan-cancer was analyzed with TIMER. (*p < 0.05, **p < 0.01, ***p < 0.001). **(B, C)** The expression and prognostic value of the IGF2BP2 gene in HNSCC was analyzed with GEPIA2. (HR, hazard ratio) **(D)** Kaplan–Meier survival curves illustrate the difference in OS based on the expression level of IGF2BP2.

Based on the above analysis, we next selected IGF2BP2 for the multivariate analysis of 24 m^6^A regulatory genes. The expression of the IGF2BP2 gene in pan-cancer was examined with TIMER (**Figure 3A**), and we verified the expression and prognostic value of the IGF2BP2 gene in HNSCC using GEPIA2 (**Figures 3B, C**). Kaplan–Meier curve analysis also indicated that patients with higher IGF2BP2 expression experienced significantly worse OS than their counterparts (**Figure 3D**).

### Genetic Alterations in m^6^A Regulatory Genes and Mutation, CNV, and Methylation Analyses of IGF2BP2

The incidence of genetic alterations in the 24 m^6^A regulators in HNSCC (TCGA, Firehose Legacy) was analyzed by using the cBioPortal tool. Twenty-four m^6^A methylation regulators showed genetic alterations in 227 (45%) queried patients/samples (504 patients/samples). The most common genetic alterations were amplification and deep deletion mutations. In our analysis, IGF2BP2 exhibited the highest mutation frequency (an alteration rate of 20%), followed by KIAA1429 (VIRMA) (an alteration rate of 8%), while both METTL14 and YTHDF2 showed few mutations in HNSCC samples (**Supplementary Material: Figure S3A**). Furthermore, the potential mechanism of dysregulated IGF2BP2 was studied because it was identified as an independent prognostic factor for OS. In the UCSC Xena database, 604 samples from the TCGA-HNSC dataset were analyzed, which revealed that IGF2BP2 expression was associated with CNV and partial DNA methylation sites but not with somatic mutations (**Supplementary Material: Figure S3B**). In conclusion, CNV and DNA methylation may give rise to the abnormal upregulation of IGF2BP2 in HNSCC.

### Prediction of IGF2BP2 Transcription Factors

**Figure 4:**
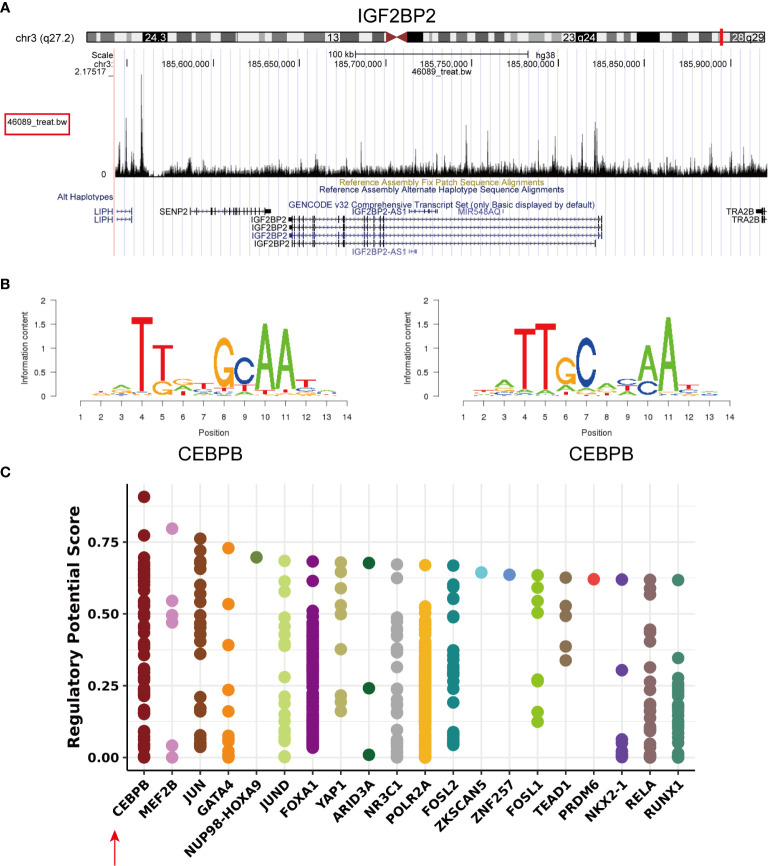
Transcription factor prediction of IGF2BP2. **(A)** Visual presentation of the high binding peak of the transcription factor CEBPB in the promoter region of IGF2BP2 **(B)** Representative sequence logo of the binding specificity of the transcription factor CEBPB queried from Cistrome. **(C)** Static plot of the top 20 factors that regulate IGF2BP2. Y axis shows the RP score. X axis shows different factors. Dots on the x axis line indicate the same factor.

To identify the mechanisms controlling the tumorigenesis and poor prognosis of HNSCC at the transcriptional level, PROMO and Cistrome Data Browser were used to predict the transcription factor binding sites for IGF2BP2 (**Supplementary Material: Table S4**). The transcription factor CCAAT/enhancer-binding protein beta (CEBPB; C/EBP-β) was obtained after taking the intersections. IGF2BP2 (chr3:185643129-185821141) was found to be a putative target (score=2.843) of the transcription factor CEBPB by searching the ChIPseq results (GSM1010802) in Cistrome DB: 46089 (35) (**Figure 4A**). The same result was validated in the hTFtarget database (http://bioinfo.life.hust.edu.cn/hTFtarget/) (36). The motif of the transcription factor CEBPB is shown in **Figure 4B** (ID: MC00121, hits: 3187, cutoff: 6.012, z score: -107.2, -10log(pval): 690.776). A statistical plot showed the top 20 factors that regulate IGF2BP2 queried from Cistrome (**Figure 4C**), and CEBPB ranked first. In summary, the transcription factor CEBPB is most likely the transcription factor that regulates IGF2BP2.

### IGF2BP2-Associated Genes Mediate m^6^A Modification

**Figure 5:**
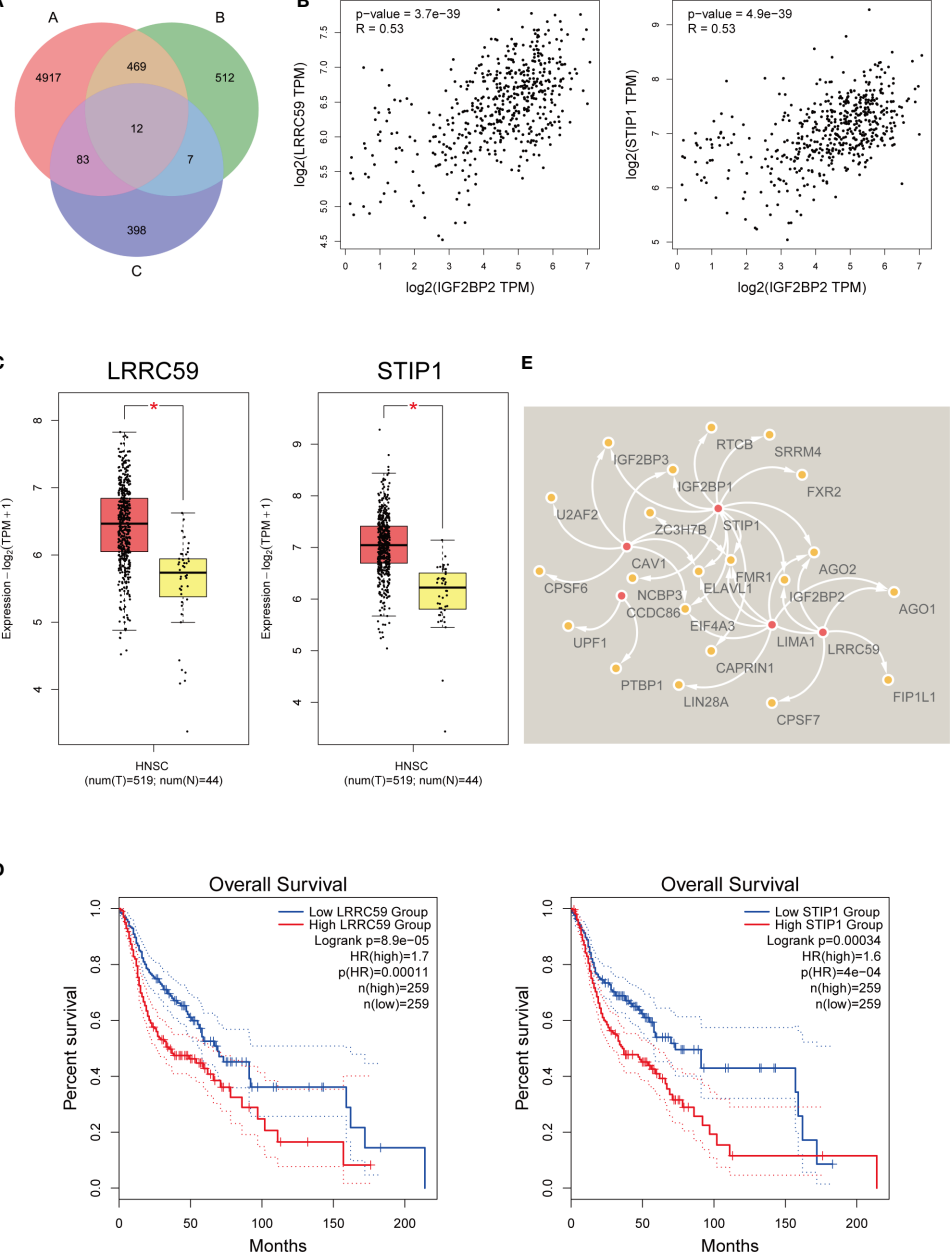
IGF2BP2-associated genes that mediate m^6^A modification. **(A)** Venn chart of IGF2BP2-related genes in HNSCC. **(B)** The correlation of LRRC59, STIP1, and IGF2BP2. **(C)** The expression of LRRC59 and STIP1 was confirmed with GEPIA2. T, tumor tissue. N, normal tissue. * means p-value < 0.01. **(D)** Kaplan-Meier survival curves illustrate the difference in OS according to the expression of LRRC59 and STIP1. **(E)** The relationships of RBPs and IGF2BP2-associated genes with the most potential.

Initially, we first collated an “A” list of dysregulated genes in HNSCC (n=5481) (log2FC cutoff = 0.58, p-value cutoff = 0.01), a “B” list of the top 1000 similar gene correlations of IGF2BP2 (n=1000), and then a “C” list of genes related to the survival of HNSCC patients (n=500). The number of genes in each list is displayed as a Venn diagram (**Figure 5A**). Twelve genes were common to all three gene sets. Therefore, they may act as IGF2BP2-associated genes and play a regulatory role in m^6^A modification. We analyzed their relationships with IGF2BP2 again. For example, we found that leucine-rich repeat-containing protein 59 (LRRC59) and stress-induced phosphoprotein 1 (STIP1) were correlated with IGF2BP2 (R=0.53 and R=0.53, respectively) (**Figure 5B**). LRRC59 and STIP1 were upregulated in HNSCC according to GEPIA2 (log2FC cutoff = 1, p-value cutoff = 0.01) (**Figure 5C**). High LRRC59 and STIP1 expression was correlated with poor OS in HNSCC patients (**Figure 5D**). It was predicted that IGF2BP2 facilitates the recruitment of RBPs to m^6^A-modified target mRNAs. By analyzing m^6^A Var, we analyzed the RBPs of potential associated genes, and their relationships were visualized with Cytoscape (37) (**Figure 5E**).

### Potential Regulatory Mechanisms of IGF2BP2

**Figure 6:**
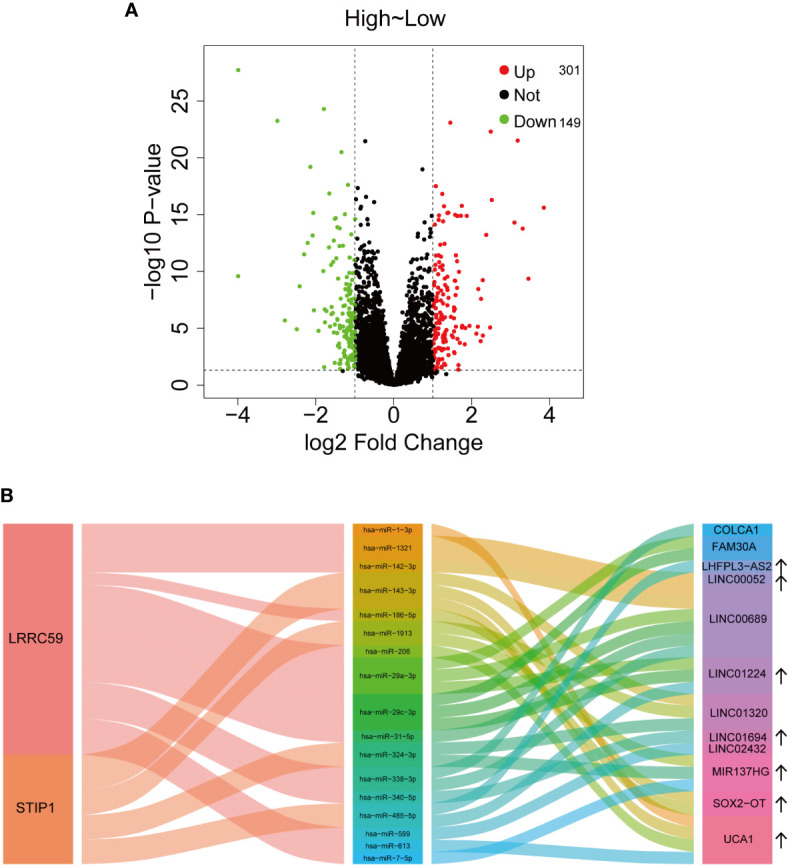
Potential regulatory mechanisms of IGF2BP2. **(A)** The dysregulated lncRNAs associated with IGF2BP2 expression. **(B)** Sankey plot illustrating the ceRNA regulatory network.

Based on the above analysis, a total of 301 upregulated and 149 downregulated lncRNAs are significantly related to IGF2BP2 expression (**Figure 6A**). Among them, some lncRNAs are associated with HNSCC. The lncRNA SOX2-OT was reported to inhibit PTEN expression to facilitate laryngeal squamous cell carcinoma (LSCC) development through EZH2-mediated H3K27me3 (38). The potential target miRNAs (at least four of seven platforms predicted) and corresponding lncRNAs were predicted with starBase v2.0 (http://starbase.sysu.edu.cn/) (39). Finally, a Sankey plot consisting of lncRNAs, microRNAs (miRNAs), and two mRNAs (LRRC59 and STIP1) was constructed to illustrate a competing endogenous RNA (ceRNA) regulatory network (**Figure 6B**).

### Unsupervised Consensus Analysis of m^6^A Regulatory Genes

**Figure 7:**
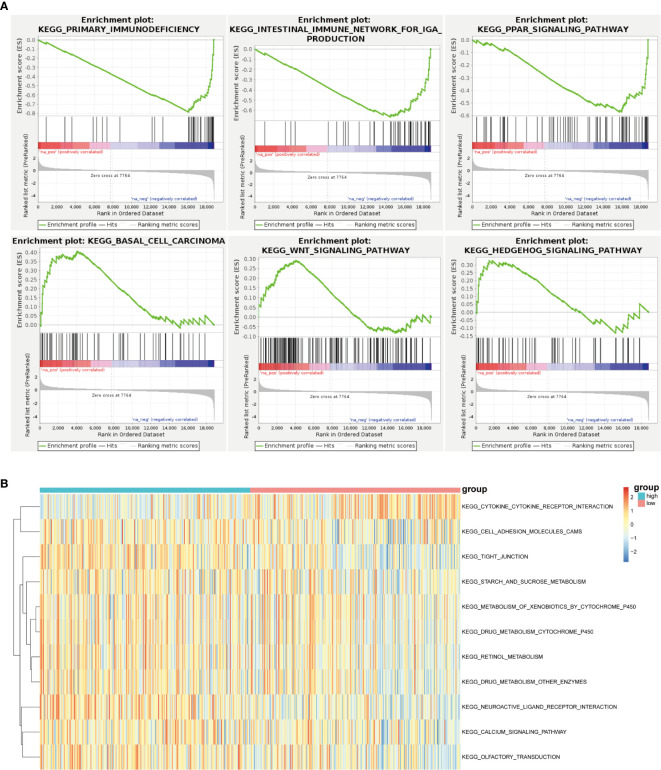
Unsupervised consensus analysis of m^6^A regulatory genes. **(A)** GSEA revealed that the IGF2BP2 group was enriched in the hallmarks malignant progression and tumor immunology. **(B)** GSVA revealed the state of biological pathways in the high and low IGF2BP2 groups. The column represents the TCGA-HNSCC samples.

With the help of GSEA, the malignant hallmarks of tumors were investigated. The results showed that the tumor hallmarks primary immunodeficiency (NES=-2.21, normalized P < 0.0001), intestinal immune network for IgA production (NES=-1.95, normalized P < 0.0001), PPAR signaling pathway (NES=-1.80, normalized P< 0.001), basal cell carcinoma (NES=1.53, normalized P =0.013), Wnt signaling pathway (NES=1. 30, normalized P < 0.001), and Hedgehog signaling pathway (NES=1. 26, normalized P =0.080) were enriched in the IGF2BP2 group (**Figure 7A**).

Furthermore, GSVA was applied to illustrate the biological behaviors among distinct m^6^A modification patterns. As shown in **Figure 7B**, the high IGF2BP2 group was markedly enriched in cell adhesion molecules, neuroactive ligand receptor interaction pathways, and calcium signaling pathways. In our study, it was concluded that low IGF2BP2 expression was enriched in the cytokine receptor interaction pathway.

Based on the above analyses, we speculate that the IGF2BP2-mediated m^6^A methylation modification has significant associations with the malignant progression and tumor immunology of HNSCC.

### The Protein Expression of m^6^A Target Genes

**Figure 8:**
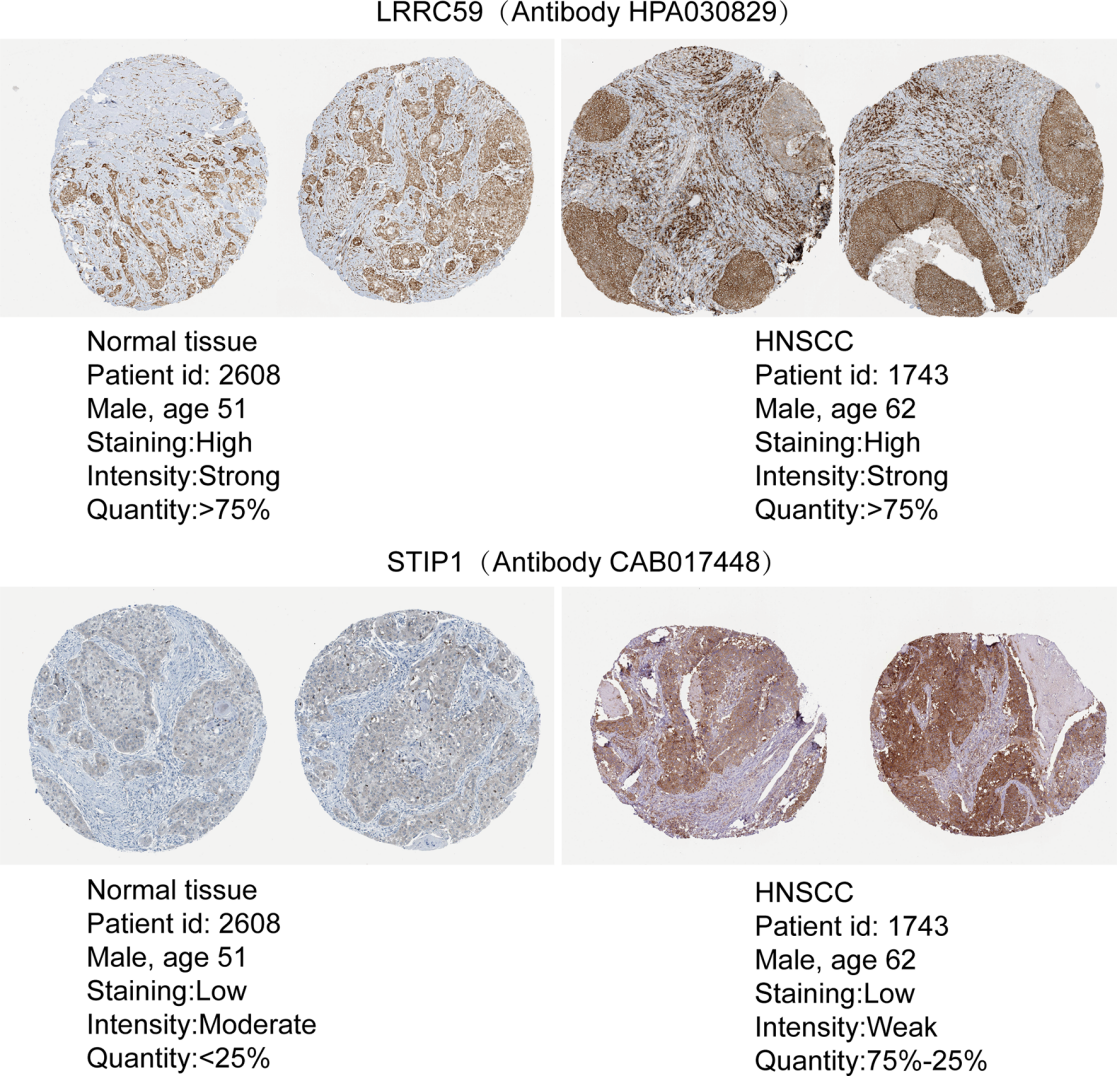
The protein expression of m^6^A target genes. A Comparison of the protein expression of LRRC59 and STIP1 in HNSCC tumor tissues and normal tissues.

To evaluate the expression differences in target genes at the protein level, immunohistochemistry data were obtained from pathological specimens from The Human Protein Atlas. We found that LRRC59 and STIP1 were dysregulated between HNSCC tumor tissues and normal tissues (**Figure 8**).

### Relationship Between the Tumor-Immune Microenvironment and mRNA Expression of IGF2BP2

**Figure 9:**
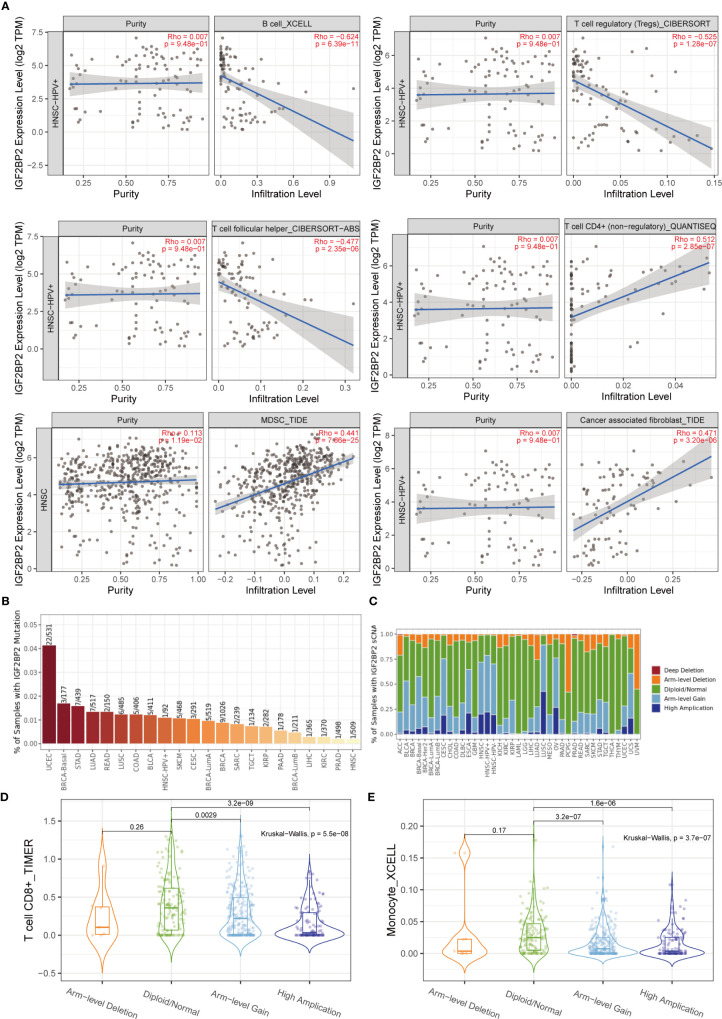
Relationship between the tumor-immune microenvironment and IGF2BP2 expression. **(A)** The relationship between IGF2BP2 and immune cell infiltration in HNSCC. **(B)** Bar plot of IGF2BP2 mutation frequency in each TCGA cancer type. **(C)** The stacked bar chart shows the relative proportions of IGF2BP2 in different sCNA states in all tumor types. **(D)** Differential T cell CD8+ infiltration level in HNSCC (TIMER). **(E)** Differential monocyte infiltration level in HNSCC (XCELL).

After determining the malignant hallmarks of IGF2BP2, the relationship between IGF2BP2 expression and tumor immune cell infiltration in cancers was explored. First, the correlation of IGF2BP2 expression and immune infiltration levels was evaluated in diverse cancer types. The relationship between IGF2BP2 and 21 kinds of immune cells indicated a negative correlation between IGF2BP2 and B cells, CD4+ T cells, follicular helper T cells, regulatory T cells (Tregs), myeloid-derived suppressor cells, and cancer-associated fibroblast cells in HNSCC (**Figure 9A**). For example, the expression level of IGF2BP2 had a negative correlation with the immune infiltration level of B cells (Spearman r = -0.624, p=6.39e-11). The mutation frequency of the IGF2BP2 gene in each TCGA cancer type is shown as a bar plot (**Figure 9B**). We found that HNSCC has the lowest IGF2BP2 mutation frequency among multiple cancer types. Therefore, IGF2BP2 gene mutations may have a weak relationship with immune cell infiltration across 22 multiple cancer types and immune cell types. The relative proportions of IGF2BP2 in different sCNA states in all tumor types are shown as a stacked bar chart (**Figure 9C**). We found that high amplification, arm-level gain, and diploidy were the most common states. It was also found that the distribution of immune cell infiltration in different sCNA states was different. For example, the differential T cell CD8+ (TIMER) and monocyte (XCELL) infiltration levels were observed in these sCNA states in HNSCC (**Figures 9D, E**).

## Discussion

The abnormal epigenetic modification of RNA is important to tumorigenesis and cancer patient prognosis. As the most common modification type in eukaryotic mRNA, m^6^A is characterized by the N6 methylation of adenosine. Its role in cancer has gradually been recognized (6). It was recently found that m^6^A methylation has an indispensable function in innate immunity and the tumor microenvironment. This study focused on the abnormal gene expression of m^6^A regulators in pan-cancer and the molecular characteristics, prognostic value, and immune characteristics of m^6^A regulators in HNSCC.

In this study, we generated a catalog of 24 genes that act as regulators of m^6^A (8 writers, 2 erasers and 14 readers). After exploring their expression landscape in pan-cancer, it was noted that most m^6^A regulatory genes were abnormally expressed in tumors in a cancer-specific manner, and all the m^6^A regulatory genes were correlated with patient OS in at least one cancer type. These results were consistent with the findings of previous studies. For instance, METTL3 was found to be upregulated in colorectal cancer (CRC), and high METTL3 expression was associated with poor OS in CRC patients (40). Similarly, IGF2BP2 was upregulated and found to be related to a poor prognosis in pancreatic cancer (41) and esophagogastric junction adenocarcinoma (42). Moreover, it was found that combined levels of METTL3 and YTHDF1 could reflect the malignancy state and prognosis of hepatocellular carcinoma (HCC) (43). Our findings showed that most m^6^A regulatory genes were upregulated or downregulated in HNSCC, suggesting that these genes might be related to the carcinogenic activity of tumor cells and/or the prognosis of HNSCC patients. Among these m^6^A regulatory genes, IGF2BP2 was upregulated in HNSCC tumor tissues, and high IGF2BP2 expression was associated with poor patient OS in seven cancer types, including HNSCC. Similar studies in other cancers, such as colorectal, pancreatic, and breast cancers, have reported similar results (44–46). We found that the expression of IGF2BP2, RBMX, and HNRNPC was correlated with various activated cancer-related pathways, providing insight into the molecular mechanisms related to tumor progression. Furthermore, m^6^A regulators are useful as biomarkers for prognostic stratification (47). Our results showed that IGF2BP2 expression was negatively correlated with the prognosis of HNSCC, so IGF2BP2 is very likely an oncogene in HNSCC. In addition, CNV and DNA methylation, but not somatic mutations, give rise to the abnormal upregulation of IGF2BP2 in HNSCC based on data from TCGA-HNSCC patients. This result further shows that epigenetic modifications are also pivotal to the aberrant expression of IGF2BP2, an epigenetic modifier (41). This research suggests that the abnormal expression of m^6^A regulatory genes such as IGF2BP2 is common in HNSCC, but relatively little information on the function of IGF2BP2 in tumorigenesis is currently available, and its precise expression and mechanisms require further investigation.

Regarding the mechanisms controlling the tumorigenesis and poor prognosis of HNSCC at the transcriptional level, we found that the transcription factor CEBPB was the most likely to bind to the promoter region of IGF2BP2. Dysregulated IGF2BP2 could also affect the associated downstream genes *via* the m^6^A modification to accelerate cancer progression (41). Our study showed that 12 genes, including LRRC59 and STIP1, may act as IGF2BP2-associated genes to play a regulatory role in the m^6^A modification. LRRC59 and STIP1 have been reported to be closely associated with the progression of human malignancies. For instance, high LRRC59 expression is closely related to poor survival in LUAD patients and an aggressive phenotype of breast cancer (48). STIP1 is also upregulated in LUAD and may be associated with the enhanced proliferation, adhesion, and migration and inhibition of apoptosis in LUAD cells *via* the JAK2/STAT3 signaling pathway and EMT (49). Similar findings have been reported in HCC and ovarian, pancreatic, renal, and gastric cancers (50, 51). Noncoding RNAs are associated with the regulation of the m^6^A modification and can further affect the malignant progression of cancer (52, 53). Interestingly, a series of abnormally expressed m^6^A-modified miRNAs or lncRNAs could perform their function by acting as ceRNAs (54, 55), suggesting potential mechanisms in the dysregulation of IGF2BP2-associated genes in HNSCC. For example, IGF2BP2 serves as a reader of m^6^A-modified lncRNA-DANCR and can stabilize DANCR, which reversely contributes to tumor stemness-like properties and the pathogenesis of pancreatic cancer (41). So, in order to predict the key factors that exist in this regulatory relationship, we derived a regulatory network of IGF2BP2-associated genes, miRNAs, and lncRNAs.

In recent years, there has been increasing interest in m^6^A modification in multiple tumor biological processes and signaling pathways not limited to the growth of tumor stem cells, neoplasia (56–58), RNA metabolism (59), and the DNA damage response after radiotherapy and chemotherapy (60). By performing GSEA, the malignant hallmarks of IGF2BP2 in HNSCC were analyzed and may include basal cell carcinoma, the Wnt signaling pathway, the Hedgehog signaling pathway, and some signaling pathways related to tumor immunology, such as primary immunodeficiency and the intestinal immune network for IgA production. GSVA showed that the high IGF2BP2 group was markedly enriched in the cell adhesion molecule CAMS, neuroactive ligand receptor interaction pathways, and the calcium signaling pathway. This study uncovered that IGF2BP2-mediated m^6^A methylation was strongly correlated with the malignant progression of HNSCC and tumor immunology signaling pathways. The subsequent assays revealed a negative correlation between IGF2BP2 and multiple tumor-infiltrating immune cells, such as B cells, CD4+ T cells, follicular helper T cells, and Tregs. Our findings may provide novel insight into immunotherapy targets in HNSCC.

However, this study also has some limitations. For example, the potential mechanisms by which IGF2BP2 is upregulated are only descriptive. In the future, more basic functional mechanistic studies will be needed to validate our findings, and the clinical application of m^6^A methyltransferase in HNSCC needs further exploration.

## Conclusions

In conclusion, by systematically analyzing the gene expression landscape of 24 m^6^A regulators in pan-cancer, IGF2BP2 was found to be highly expressed in HNSCC, is correlated with tumor progression and a low survival rate, and is involved in tumor immune invasion. More importantly, IGF2BP2 might be a promising candidate as a molecular biomarker for monitoring HNSCC development and provide ideal guidance for selecting therapeutic strategies. This study provides a supplement at the molecular level for epigenetic regulation in HNSCC and ideas for effective immunotherapy targets and strategies.

## Data Availability Statement

The original contributions presented in the study are included in the article/**Supplementary Material**. Further inquiries can be directed to the corresponding authors.

## Author Contributions

XCG and YHZ were the major contributors to the writing and revision of the manuscript. ZMZ and ZRZ collected the related references and participated in discussions. XCG and QL made substantial contributions to the conception or design of the work. QL, WTY, and HW approved the final version of the manuscript. All authors contributed to the article and approved the submitted version.

## Funding

This work was financially supported by grants from the Natural Science Foundation of Hebei Province (no. H2020201050), the National Innovation and Entrepreneurship Training Plan in 2020 (no. 202014432031), Science and Technology Capacity Improvement Projects of Hebei University of Chinese Medicine in 2019 (no. KTZ2019019), Outstanding Student Scientific Research Ability Improvement Projects of Hebei University of Chinese Medicine in 2019 (no. YXZ2019002), and Graduate Innovative Ability Training Projects of Hebei Province in 2020 (no. XCXZZBS2020002) (no. hbu2020bs003).

## Conflict of Interest

The authors declare that the research was conducted in the absence of any commercial or financial relationships that could be construed as a potential conflict of interest.

## Supplementary Material

Supplementary Figure 1 Relationships between m^6^A regulatory genes and pan-cancer. **(A)** summary of the relevance between clinical patient survival and expression of m^6^A regulators. Red dots illustrate high expression of m^6^A regulators related to poor survival, and blue dots illustrate prolonged survival. Only genes with a Kaplan-Meier log-rank test p-value < 0.05 are shown. **(B)** Heatmap represents the percentage of cancers in which the pathway may be activated or inhibited by 24 m^6^A regulators analyzed with GSCALite. Red represents the percentage of cancers whose pathways are likely activated, and blue represents the percentage of cancers whose pathways are likely inhibited.

Supplementary Figure 2 SNP and SNV mutations in m^6^A regulatory genes. **(A–C)** SNP types in 24 m^6^A regulatory genes **(D)** The mutation frequencies of 24 m^6^A regulators in 1443 patients from the TCGA pan-cancer cohort. Individual patients are represented in the column. The right bar plot shows the proportion of each variant type.

Supplementary Figure 3 Genetic alterations in m^6^A regulatory genes. **(A)** Genetic alteration profiles of 24 m^6^A regulatory genes in the TCGA dataset. The data were obtained from the cBioPortal for Cancer Genomics. **(B)** Heatmap of the correlations between IGF2BP2 and somatic mutations (MC3 gene-level nonsilent mutation), CNV, and methylation in HNSCC *via* UCSC Xena.

Supplementary Table 1 Information on thirty-three cancer types from the TCGA.

Supplementary Table 2 Correlation of IGF2BP2 expression and clinicopathologic characteristics in HNSCC.

Supplementary Table 3 Correlation of ELVAL1 and LRPPRC expression and clinicopathologic characteristics.

Supplementary Table 4 Prediction of IGF2BP2 transcription factors (based on binding to its promoter) with PROMO (dissimilarity rate =0%).

## References

[1] Michalak EM Burr ML Bannister AJ Dawson MA. The roles of DNA, RNA and histone methylation in ageing and cancer. Nat Rev Mol Cell Biol (2019) 20:573–89. 10.1038/s41580-019-0143-1 31270442

[2] Koch A Joosten SC Feng Z de Ruijter TC Draht MX Melotte V. Analysis of DNA methylation in cancer: location revisited. Nat Rev Clin Oncol (2018) 15:459–66. 10.1038/s41571-018-0004-4 29666440

[3] Lan Q Liu PY Haase J Bell JL Hüttelmaier S Liu T. The Critical Role of RNA m(6)A Methylation in Cancer. Cancer Res (2019) 79:1285–92. 10.1158/0008-5472.Can-18-2965 30894375

[4] Zhang Y Geng X Li Q Xu J Tan Y Xiao M. m6A modification in RNA: biogenesis, functions and roles in gliomas. J Exp Clin Cancer Res (2020) 39:192. 10.1186/s13046-020-01706-8 32943100 PMC7500025

[5] Delaunay S Frye M. RNA modifications regulating cell fate in cancer. Nat Cell Biol (2019) 21:552–9. 10.1038/s41556-019-0319-0 31048770

[6] Deng X Su R Weng H Huang H Li Z Chen J. RNA N(6)-methyladenosine modification in cancers: current status and perspectives. Cell Res (2018) 28:507–17. 10.1038/s41422-018-0034-6 PMC5951805 29686311

[7] Frye M Harada BT Behm M He C. RNA modifications modulate gene expression during development. Science (2018) 361:1346–9. 10.1126/science.aau1646 PMC6436390 30262497

[8] Li A Chen YS Ping XL Yang X Xiao W Yang Y. Cytoplasmic m(6)A reader YTHDF3 promotes mRNA translation. Cell Res (2017) 27:444–7. 10.1038/cr.2017.10 PMC5339832 28106076

[9] Tang C Klukovich R Peng H Wang Z Yu T Zhang Y. ALKBH5-dependent m6A demethylation controls splicing and stability of long 3’-UTR mRNAs in male germ cells. Proc Natl Acad Sci U S A (2018) 115:E325–33. 10.1073/pnas.1717794115 29279410 PMC5777073

[10] Ries RJ Zaccara S Klein P Olarerin-George A Namkoong S Pickering BF. m(6)A enhances the phase separation potential of mRNA. Nature (2019) 571:424–8. 10.1038/s41586-019-1374-1 PMC6662915 31292544

[11] Tang C Xie Y Yu T Liu N Wang Z Woolsey RJ. m(6)A-dependent biogenesis of circular RNAs in male germ cells. Cell Res (2020) 30:211–28. 10.1038/s41422-020-0279-8 PMC7054367 32047269

[12] Xu K Yang Y Feng GH Sun BF Chen JQ Li YF. Mettl3-mediated m(6)A regulates spermatogonial differentiation and meiosis initiation. Cell Res (2017) 27:1100–14. 10.1038/cr.2017.100 PMC5587845 28809392

[13] Zhang C Chen Y Sun B Wang L Yang Y Ma D. m(6)A modulates haematopoietic stem and progenitor cell specification. Nature (2017) 549:273–6. 10.1038/nature23883 28869969

[14] Zheng Q Hou J Zhou Y Li Z Cao X. The RNA helicase DDX46 inhibits innate immunity by entrapping m(6)A-demethylated antiviral transcripts in the nucleus. Nat Immunol (2017) 18:1094–103. 10.1038/ni.3830 28846086

[15] Liu N Pan T. N6-methyladenosine–encoded epitranscriptomics. Nat Struct Mol Biol (2016) 23:98–102. 10.1038/nsmb.3162 26840897

[16] Ding C Zou Q Ding J Ling M Wang W Li H. Increased N6-methyladenosine causes infertility is associated with FTO expression. J Cell Physiol (2018) 233:7055–66. 10.1002/jcp.26507 PMC13482491 29384212

[17] Chen X Yu C Guo M Zheng X Ali S Huang H. Down-Regulation of m6A mRNA Methylation Is Involved in Dopaminergic Neuronal Death. ACS Chem Neurosci (2019) 10:2355–63. 10.1021/acschemneuro.8b00657 30835997

[18] Geula S Moshitch-Moshkovitz S Dominissini D Mansour AA Kol N Salmon-Divon M. Stem cells. m6A mRNA methylation facilitates resolution of naïve pluripotency toward differentiation. Science (2015) 347:1002–6. 10.1126/science.1261417 25569111

[19] Li Y Xiao J Bai J Tian Y Qu Y Chen X. Molecular characterization and clinical relevance of m(6)A regulators across 33 cancer types. Mol Cancer (2019) 18:137. 10.1186/s12943-019-1066-3 31521193 PMC6744659

[20] He L Li H Wu A Peng Y Shu G Yin G. Functions of N6-methyladenosine and its role in cancer. Mol Cancer (2019) 18:176. 10.1186/s12943-019-1109-9 31801551 PMC6892141

[21] Wang S Sun C Li J Zhang E Ma Z Xu W. Roles of RNA methylation by means of N(6)-methyladenosine (m(6)A) in human cancers. Cancer Lett (2017) 408:112–20. 10.1016/j.canlet.2017.08.030 28867248

[22] Chen M Wong CM. The emerging roles of N6-methyladenosine (m6A) deregulation in liver carcinogenesis. Mol Cancer (2020) 19:44. 10.1186/s12943-020-01172-y 32111216 PMC7047367

[23] Dixit D Prager BC Gimple RC Poh HX Wang Y Wu Q. The RNA m6A reader YTHDF2 maintains oncogene expression and is a targetable dependency in glioblastoma stem cells. Cancer Discovery (2020) 11:480–99. 10.1158/2159-8290.Cd-20-0331 PMC8110214 33023892

[24] Rai V Aggarwal SK Verma SS Awasthee N Dhasmana A Aggarwal S. Epoxyazadiradione exhibit activities in head and neck squamous cell carcinoma by targeting multiple pathways. Apoptosis (2020) 25:763–82. 10.1007/s10495-020-01633-1 32894380

[25] Bray F Ferlay J Soerjomataram I Siegel RL Torre LA Jemal A. Global cancer statistics 2018: GLOBOCAN estimates of incidence and mortality worldwide for 36 cancers in 185 countries. CA Cancer J Clin (2018) 68:394–424. 10.3322/caac.21492 30207593

[26] Carlisle JW Steuer CE Owonikoko TK Saba NF. An update on the immune landscape in lung and head and neck cancers. CA Cancer J Clin (2020) 70:505–17. 10.3322/caac.21630 32841388

[27] Zhao Q Zhao Y Hu W Zhang Y Wu X Lu J. m(6)A RNA modification modulates PI3K/Akt/mTOR signal pathway in Gastrointestinal Cancer. Theranostics (2020) 10:9528–43. 10.7150/thno.42971 PMC7449908 32863943

[28] Zhang B Wu Q Li B Wang D Wang L Zhou YL. m(6)A regulator-mediated methylation modification patterns and tumor microenvironment infiltration characterization in gastric cancer. Mol Cancer (2020) 19:53. 10.1186/s12943-020-01170-0 32164750 PMC7066851

[29] Colaprico A Silva TC Olsen C Garofano L Cava C Garolini D. TCGAbiolinks: an R/Bioconductor package for integrative analysis of TCGA data. Nucleic Acids Res (2016) 44:e71. 10.1093/nar/gkv1507 26704973 PMC4856967

[30] Li T Fu J Zeng Z Cohen D Li J Chen Q. TIMER2.0 for analysis of tumor-infiltrating immune cells. Nucleic Acids Res (2020) 48:W509–w514. 10.1093/nar/gkaa407 32442275 PMC7319575

[31] Liu CJ Hu FF Xia MX Han L Zhang Q Guo AY. GSCALite: a web server for gene set cancer analysis. Bioinformatics (2018) 34:3771–2. 10.1093/bioinformatics/bty411 29790900

[32] Subramanian A Tamayo P Mootha VK Mukherjee S Ebert BL Gillette MA. Gene set enrichment analysis: a knowledge-based approach for interpreting genome-wide expression profiles. Proc Natl Acad Sci U S A (2005) 102:15545–50. 10.1073/pnas.0506580102 PMC1239896 16199517

[33] Liberzon A Birger C Thorvaldsdóttir H Ghandi M Mesirov JP Tamayo P. The Molecular Signatures Database (MSigDB) hallmark gene set collection. Cell Syst (2015) 1:417–25. 10.1016/j.cels.2015.12.004 PMC4707969 26771021

[34] Hänzelmann S Castelo R Guinney J. GSVA: gene set variation analysis for microarray and RNA-seq data. BMC Bioinf (2013) 14:7. 10.1186/1471-2105-14-7 PMC3618321 23323831

[35] Gertz J Savic D Varley KE Partridge EC Safi A Jain P. Distinct properties of cell-type-specific and shared transcription factor binding sites. Mol Cell (2013) 52:25–36. 10.1016/j.molcel.2013.08.037 24076218 PMC3811135

[36] Zhang Q Liu W Zhang HM Xie GY Miao YR Xia M. hTFtarget: A Comprehensive Database for Regulations of Human Transcription Factors and Their Targets. Genomics Proteomics Bioinf (2020) 18:120–8. 10.1016/j.gpb.2019.09.006 PMC7647694 32858223

[37] Shannon P Markiel A Ozier O Baliga NS Wang JT Ramage D. Cytoscape: a software environment for integrated models of biomolecular interaction networks. Genome Res (2003) 13:2498–504. 10.1101/gr.1239303 PMC403769 14597658

[38] Tai Y Ji Y Liu F Zang Y Xu D Ma S. Long noncoding RNA SOX2-OT facilitates laryngeal squamous cell carcinoma development by epigenetically inhibiting PTEN via methyltransferase EZH2. IUBMB Life (2019) 71:1230–9. 10.1002/iub.2026 30811870

[39] Li JH Liu S Zhou H Qu LH Yang JH. starBase v2.0: decoding miRNA-ceRNA, miRNA-ncRNA and protein-RNA interaction networks from large-scale CLIP-Seq data. Nucleic Acids Res (2014) 42:D92–97. 10.1093/nar/gkt1248 PMC3964941 24297251

[40] Peng W Li J Chen R Gu Q Yang P Qian W. Upregulated METTL3 promotes metastasis of colorectal Cancer via miR-1246/SPRED2/MAPK signaling pathway. J Exp Clin Cancer Res (2019) 38:393. 10.1186/s13046-019-1408-4 31492150 PMC6729001

[41] Hu X Peng WX Zhou H Jiang J Zhou X Huang D. IGF2BP2 regulates DANCR by serving as an N6-methyladenosine reader. Cell Death Differ (2020) 27:1782–94. 10.1038/s41418-019-0461-z PMC7244758 31804607

[42] Tang W Chen S Liu J Liu C Wang Y Kang M. Investigation of IGF1, IGF2BP2, and IGFBP3 variants with lymph node status and esophagogastric junction adenocarcinoma risk. J Cell Biochem (2019) 120:5510–8. 10.1002/jcb.27834 PMC6587846 30335898

[43] Zhou Y Yin Z Hou B Yu M Chen R Jin H. Expression profiles and prognostic significance of RNA N6-methyladenosine-related genes in patients with hepatocellular carcinoma: evidence from independent datasets. Cancer Manag Res (2019) 11:3921–31. 10.2147/cmar.S191565 PMC6503205 31118805

[44] Chen S Qiu H Liu C Wang Y Tang W Kang M. Relationship between IGF2BP2 and IGFBP3 polymorphisms and susceptibility to non-small-cell lung cancer: a case-control study in Eastern Chinese Han population. Cancer Manag Res (2018) 10:2965–75. 10.2147/cmar.S169222 PMC6118282 30214291

[45] McMullen ER Gonzalez ME Skala SL Tran M Thomas D Djomehri SI. CCN6 regulates IGF2BP2 and HMGA2 signaling in metaplastic carcinomas of the breast. Breast Cancer Res Treat (2018) 172:577–86. 10.1007/s10549-018-4960-2 PMC6424355 30220054

[46] Xu X Yu Y Zong K Lv P Gu Y. Up-regulation of IGF2BP2 by multiple mechanisms in pancreatic cancer promotes cancer proliferation by activating the PI3K/Akt signaling pathway. J Exp Clin Cancer Res (2019) 38:497. 10.1186/s13046-019-1470-y 31852504 PMC6921559

[47] Du J Hou K Mi S Ji H Ma S Ba Y. Malignant Evaluation and Clinical Prognostic Values of m6A RNA Methylation Regulators in Glioblastoma. Front Oncol (2020) 10:208. 10.3389/fonc.2020.00208 32211315 PMC7075451

[48] Li D Xing Y Tian T Guo Y Qian J. Overexpression of LRRC59 Is Associated with Poor Prognosis and Promotes Cell Proliferation and Invasion in Lung Adenocarcinoma. Onco Targets Ther (2020) 13:6453–63. 10.2147/ott.S245336 PMC7342457 32753886

[49] Guo X Yan Z Zhang G Wang X Pan Y Huang M. STIP1 Regulates Proliferation and Migration of Lung Adenocarcinoma Through JAK2/STAT3 Signaling Pathway. Cancer Manag Res (2019) 11:10061–72. 10.2147/cmar.S233758 PMC6890180 31819639

[50] Luo X Liu Y Ma S Liu L Xie R Li M. STIP1 is over-expressed in hepatocellular carcinoma and promotes the growth and migration of cancer cells. Gene (2018) 662:110–7. 10.1016/j.gene.2018.03.076 29596884

[51] Jing Y Liang W Liu J Zhang L Wei J Zhu Y. Stress-induced phosphoprotein 1 promotes pancreatic cancer progression through activation of the FAK/AKT/MMP signaling axis. Pathol Res Pract (2019) 215:152564. 10.1016/j.prp.2019.152564 31547977

[52] Dai F Wu Y Lu Y An C Zheng X Dai L. Crosstalk between RNA m(6)A Modification and Non-coding RNA Contributes to Cancer Growth and Progression. Mol Ther Nucleic Acids (2020) 22:62–71. 10.1016/j.omtn.2020.08.004 32911345 PMC7486578

[53] Geng X Zhang Y Li Q Xi W Yu W Shi L. Screening and functional prediction of differentially expressed circular RNAs in human glioma of different grades. Aging (Albany NY) (2020) 13:1989–2014. 10.18632/aging.202192 33323543 PMC7880344

[54] Geng X Jia Y Zhang Y Shi L Li Q Zang A. Circular RNA: biogenesis, degradation, functions and potential roles in mediating resistance to anticarcinogens. Epigenomics (2020) 12:267–83. 10.2217/epi-2019-0295 31808351

[55] Geng X Lin X Zhang Y Li Q Guo Y Fang C. Exosomal circular RNA sorting mechanisms and their function in promoting or inhibiting cancer. Oncol Lett (2020) 19:3369–80. 10.3892/ol.2020.11449 PMC7114721 32269609

[56] Cui Q Shi H Ye P Li L Qu Q Sun G. m(6)A RNA Methylation Regulates the Self-Renewal and Tumorigenesis of Glioblastoma Stem Cells. Cell Rep (2017) 18:2622–34. 10.1016/j.celrep.2017.02.059 PMC5479356 28297667

[57] Pan Y Ma P Liu Y Li W Shu Y. Multiple functions of m(6)A RNA methylation in cancer. J Hematol Oncol (2018) 11:48. 10.1186/s13045-018-0590-8 29587823 PMC5870302

[58] Chai RC Wu F Wang QX Zhang S Zhang KN Liu YQ. m(6)A RNA methylation regulators contribute to malignant progression and have clinical prognostic impact in gliomas. Aging (Albany NY) (2019) 11:1204–25. 10.18632/aging.101829 PMC6402513 30810537

[59] Dai D Wang H Zhu L Jin H Wang X. N6-methyladenosine links RNA metabolism to cancer progression. Cell Death Dis (2018) 9:124. 10.1038/s41419-017-0129-x 29374143 PMC5833385

[60] Xiang Y Laurent B Hsu CH Nachtergaele S Lu Z Sheng W. RNA m(6)A methylation regulates the ultraviolet-induced DNA damage response. Nature (2017) 543:573–6. 10.1038/nature21671 PMC5490984 28297716

