# Supplementary figures and images for: Molecular Characteristics, Prognostic Value, and Immune Characteristics of m6A Regulators Identified in Head and Neck Squamous Cell Carcinoma
Source: Front Oncol. 2021 Mar 18;11:629718. doi: 10.3389/fonc.2021.629718 (PMC8014089; doi:10.3389/fonc.2021.629718)

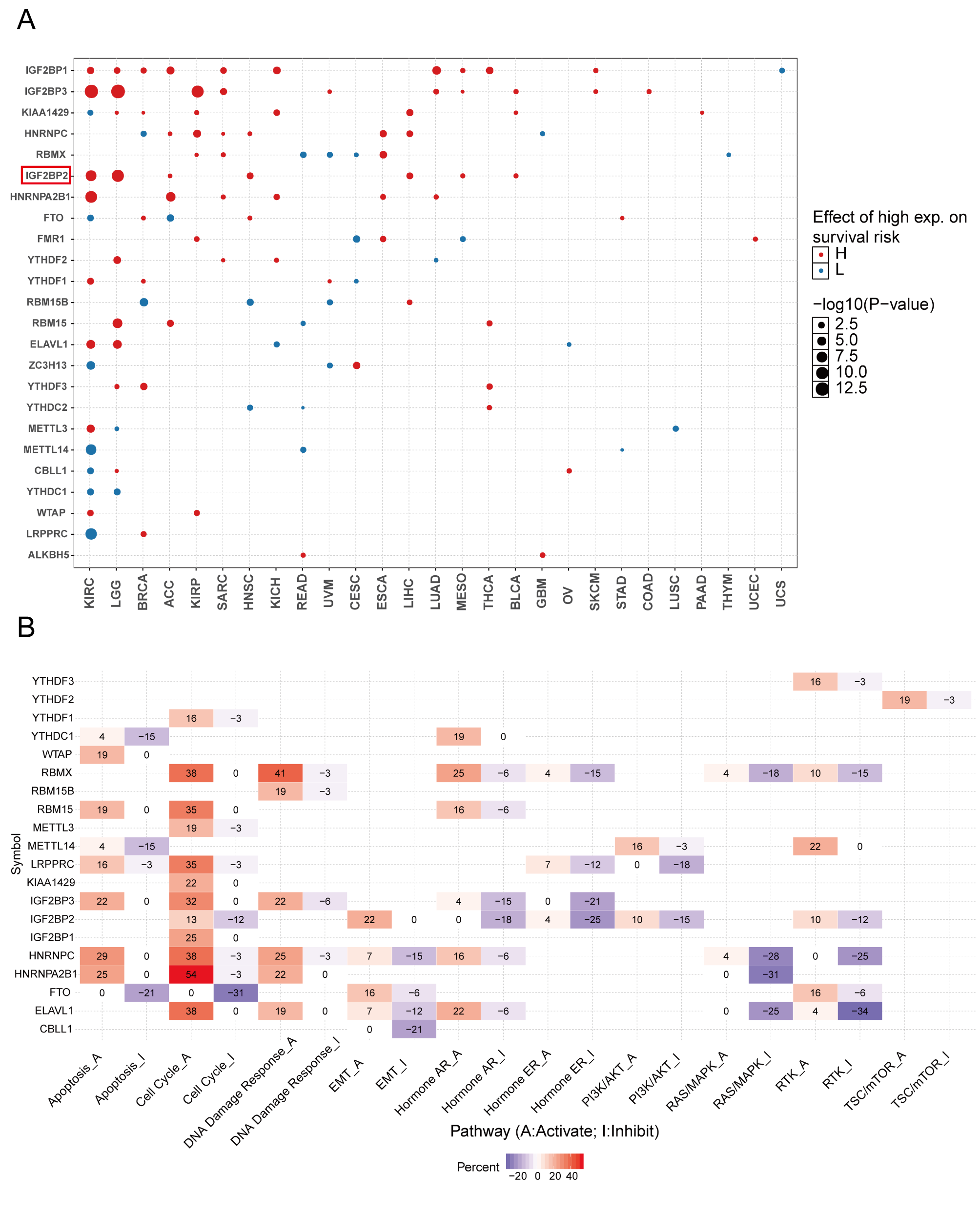

Supplement: Supplementary Figure 1 — Relationships between m6A regulatory genes and pan-cancer. (A) summary of the relevance between clinical patient survival and expression of m6A regulators. Red dots illustrate high expression of m6A regulators related to poor survival, and blue dots illustrate prolonged survival. Only genes with a Kaplan-Meier log-rank test p-value < 0.05 are shown. (B) Heatmap represents the percentage of cancers in which the pathway may be activated or inhibited by 24 m6A regulators analyzed with GSCALite. Red represents the percentage of cancers whose pathways are likely activated, and blue represents the percentage of cancers whose pathways are likely inhibited. [file Image_1.tif]

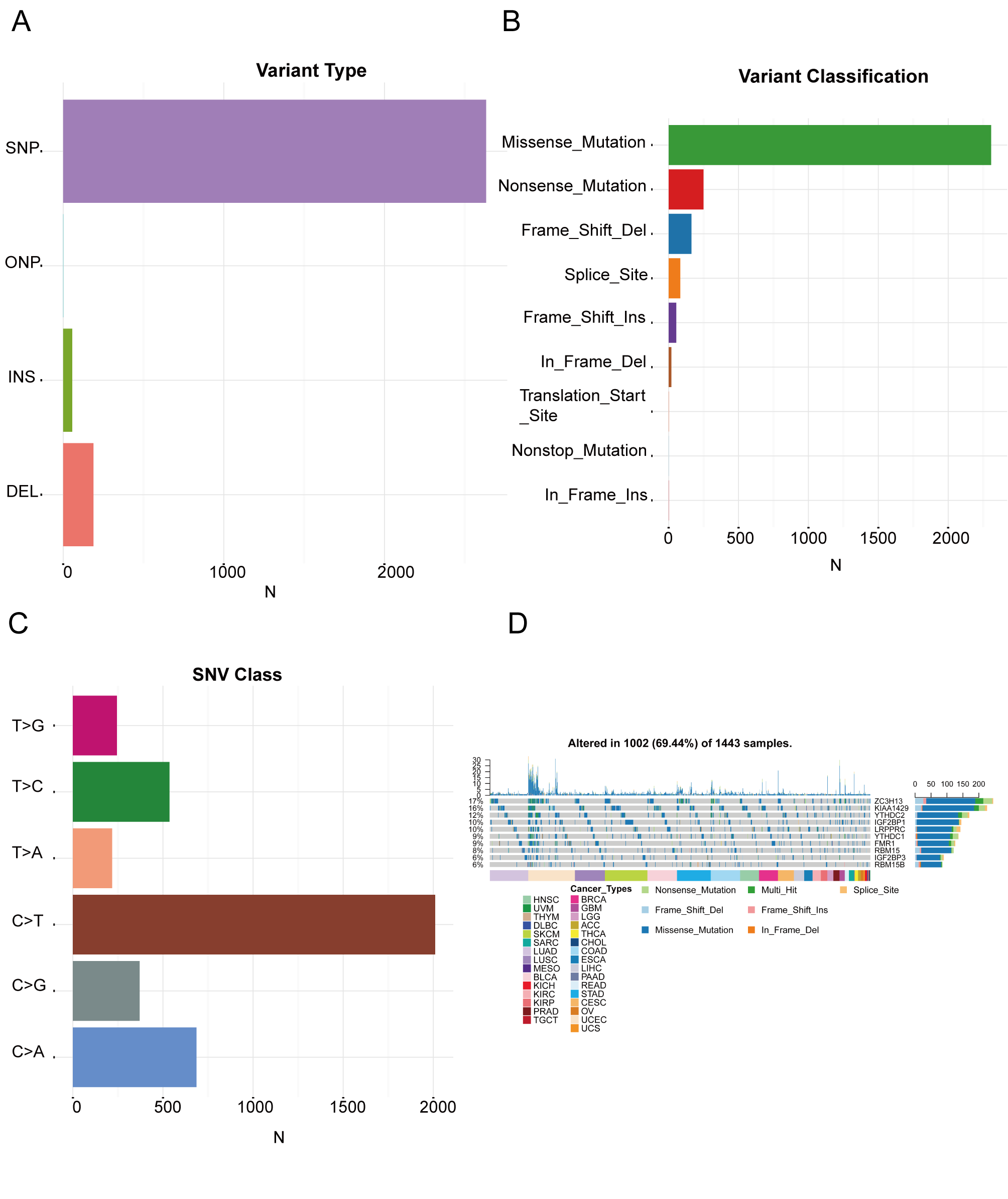

Supplement: Supplementary Figure 2 — SNP and SNV mutations in m6A regulatory genes. (A–C) SNP types in 24 m6A regulatory genes (D) The mutation frequencies of 24 m6A regulators in 1443 patients from the TCGA pan-cancer cohort. Individual patients are represented in the column. The right bar plot shows the proportion of each variant type. [file Image_2.tif]

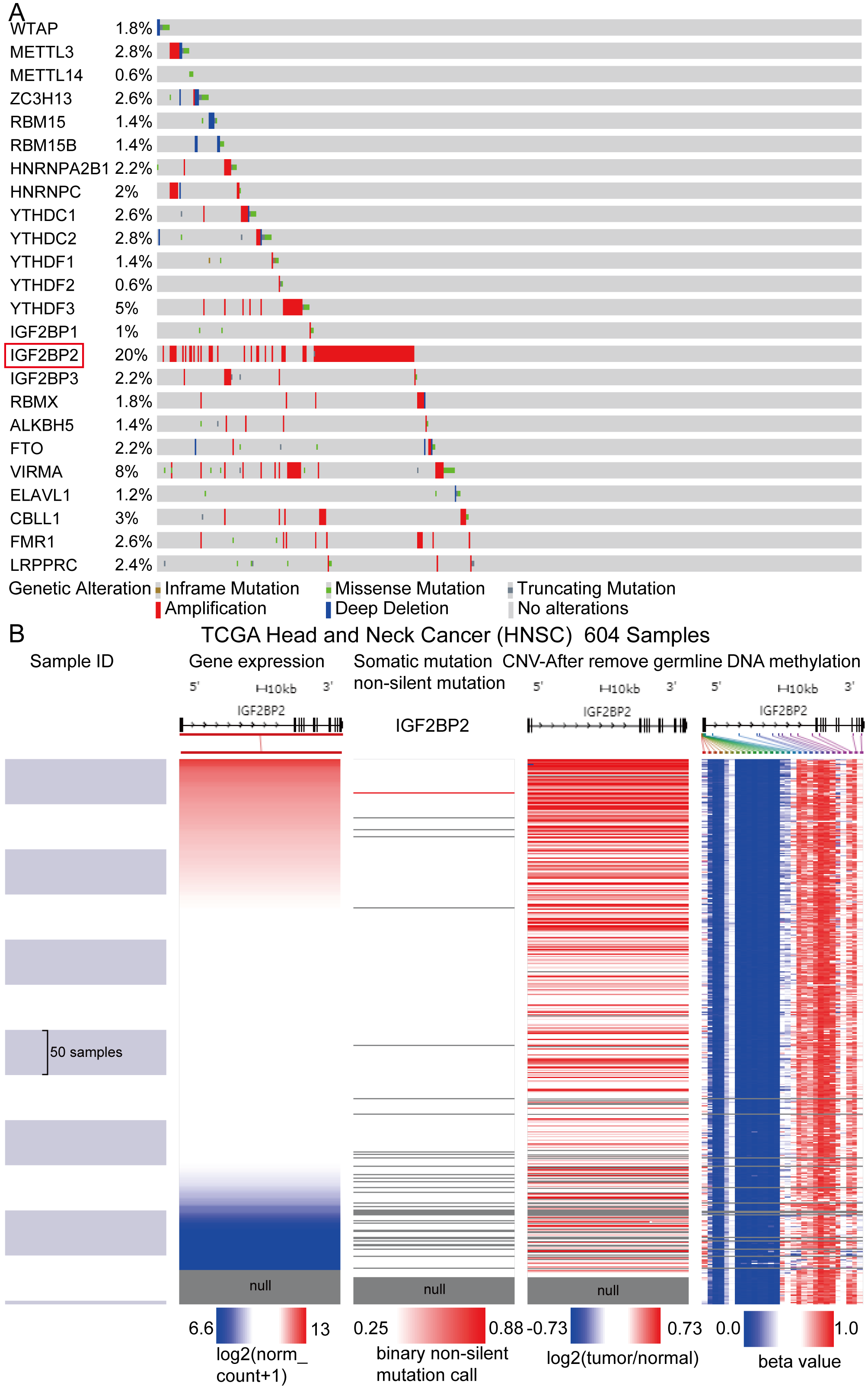

Supplement: Supplementary Figure 3 — Genetic alterations in m6A regulatory genes. (A) Genetic alteration profiles of 24 m6A regulatory genes in the TCGA dataset. The data were obtained from the cBioPortal for Cancer Genomics. (B) Heatmap of the correlations between IGF2BP2 and somatic mutations (MC3 gene-level nonsilent mutation), CNV, and methylation in HNSCC via UCSC Xena. [file Image_3.tif]
